# [Retracted] MicroRNA‑19b inhibits proliferation of gastric cancer cells by targeting B‑cell CLL/lymphoma 3

**DOI:** 10.3892/or.2023.8541

**Published:** 2023-04-03

**Authors:** Huan Wang, Mei Xiong, Yongwu Hu, Yusheng Sun, Qing Ma

Oncol Rep 36: 2079–2086, 2016; DOI: 10.3892/or.2016.5029

Following the publication of the above paper, it was drawn to the Editors' attention by a concerned reader that one of the tumor images shown in Fig. 4A was strikingly similar to a tumor image appearing in a pair of other articles that had been written by different authors at different research institutes. Owing to the fact that the contentious data in the above article had already been published elsewhere prior to its submission to *Oncology Reports*, the Editor has decided that this paper should be retracted from the Journal. The authors were asked for an explanation to account for these concerns, but the Editorial Office did not receive a reply. The Editor apologizes to the readership for any inconvenience caused.

